# Palliative medicine in 2050: how will people live the last part of life?

**DOI:** 10.1016/j.fhj.2026.100512

**Published:** 2026-03-27

**Authors:** Donna Wakefield, Sarah Perrott, George Turner, Libby Sallnow

**Affiliations:** a Wolfson Palliative Care Research Centre, Hull York Medical School, University of Hull, Hull, UK; b Health Inequalities Team, Population Health Sciences Institute, Newcastle University, Newcastle, UK; c North Tees & Hartlepool NHS Foundation Trust, Stockton-on-Tees, UK; d Marie Curie Palliative Care Research Department, University College London (UCL), London, UK

**Keywords:** Palliative care, End of life care, Inequalities, Data, Technology

## Abstract

With life expectancies rising as health-related suffering grows, and efforts to extend the human lifespan unfold against a backdrop of rising health inequalities, the landscape and nature of dying in 2050 is uncertain. Recognising this uncertainty, we have chosen to take an optimistic perspective about what 2050 may hold at the end of life, exploring not only the challenges, but also the emerging possibilities that may shape our dying, caring and grieving. This vision is primarily based on our experiences as clinicians based in the UK, but has potential to be realised more widely.

## Looking back before moving forward

**Fig. 1:**
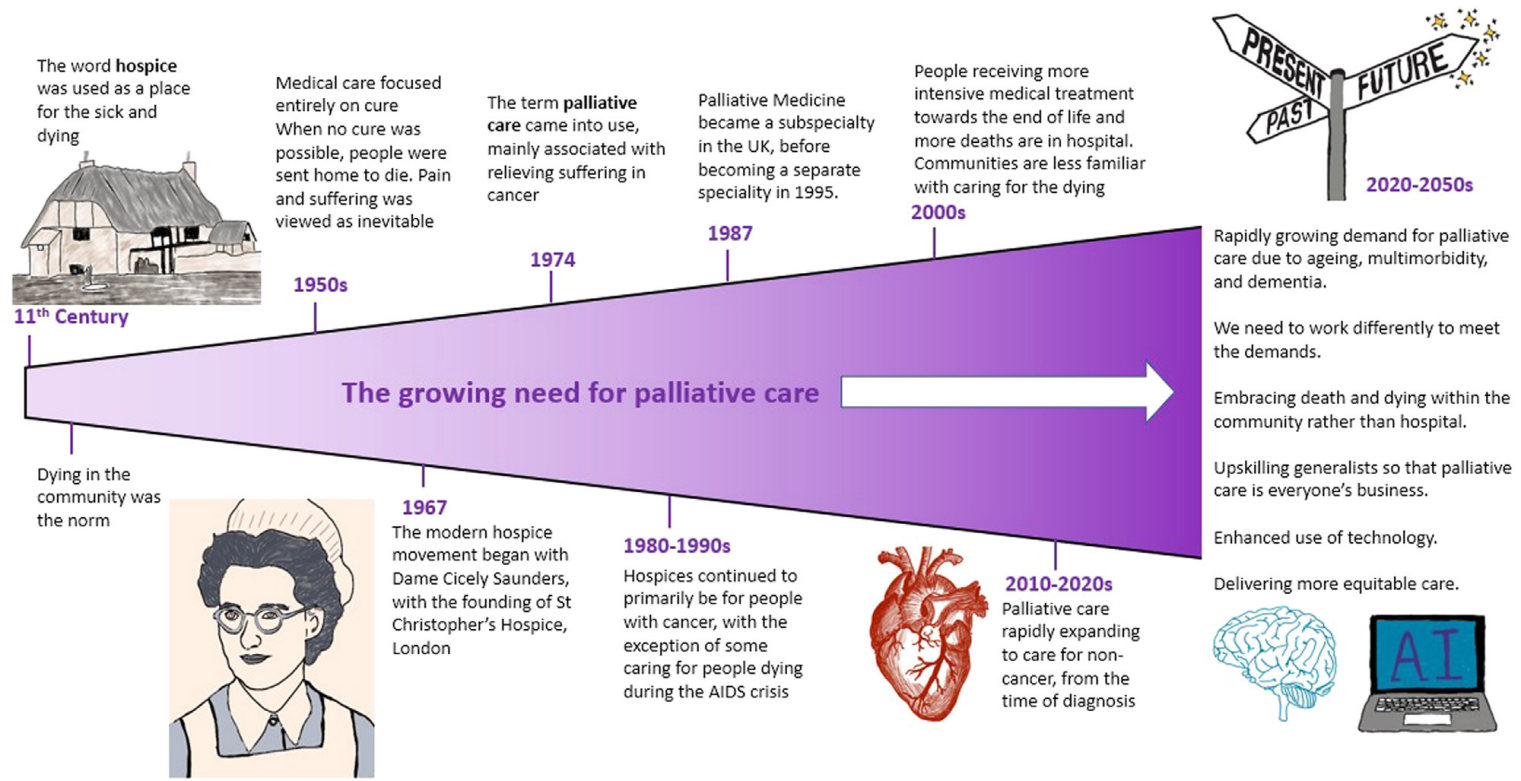
Looking back to move forward: palliative care past, present and to future.

Over the past 50 years, significant investment in science has enabled ground-breaking medical advances, curing diseases and extending life. Too little investment has been made in improving the quality of the time that people have left in the final part of their lives. It appears that the pursuit of extending life has taken priority over ensuring symptom control and comfort. In healthcare, death is often viewed as a failing, rather than a natural and unavoidable consequence of living. In many countries, such as the UK, USA, Australia and other countries in Western Europe, death has become increasingly medicalised, with families and communities unfamiliar with normal dying and often a fear of talking about it. In the UK, in the early 20th century, around 80% of people died at home, cared for by their families. A gradual shift over the course of the 20th century led to death in hospital becoming the new social norm, often with intensive medical interventions up until the point of death.^1^ Now in the early 21st century, only around 25–30% of people die at home, with families often on the sidelines, conversation about death stifled and lined with euphemisms. In this article, we look to the future and consider how palliative and end-of-life care may continue to evolve over the coming decades, approaching 2050. Fig. 1 shows key milestones: from the rise of the modern hospice movement in 1967, to the establishment of palliative medicine as a specialty in 1987, to the ongoing growth in demand and expansion of palliative care over time.

## Palliative care as everyone’s business

**Fig. 2:**
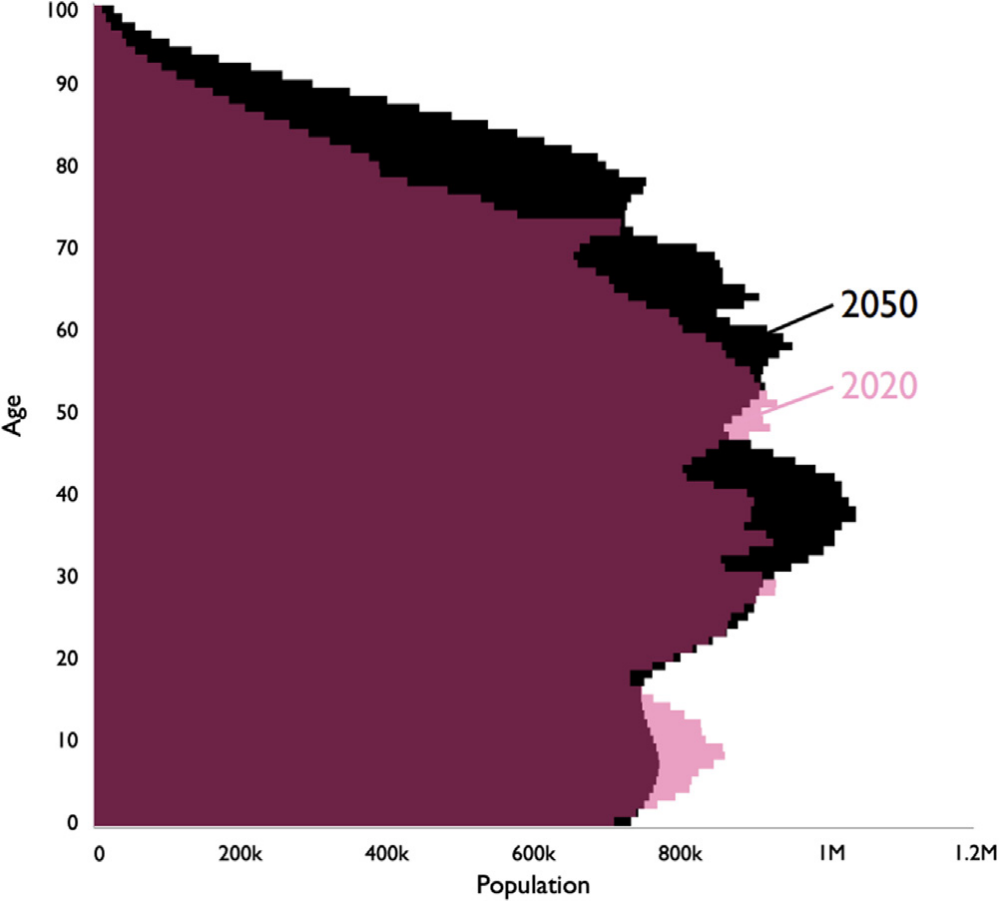
Population pyramid comparing UK population by age in 2020 (pink) and projection for 2050^7^ (black) (burgundy shows area of overlap).

Palliative care is a patient-centred approach focused on improving quality of life for people with life-limiting disease, through symptom relief and support for the patient and their family. Palliative care, including supporting straightforward end-of-life care, should be a normal part of high-quality care delivered by all clinicians, with only patients with the most complex needs requiring specialist palliative care.^2^ The need for non-specialist palliative care support for patients will become increasingly important as we approach 2050. Currently, around 75% of people approaching the end of life would benefit from palliative care. However, with an ageing population (Fig. 2) and increasing burden of multimorbidity, dementia and cancer, the need for palliative care is projected to rise by 25% even by 2040 (an estimated 160,000/year more people needing palliative care in England and Wales alone).^3^ It would be impossible for specialist palliative care services to meet this rapidly growing demand and so all clinicians, regardless of specialty, will need to deliver palliative and end-of-life care within their setting. By 2050, the goal is that high-quality education and training have enabled all clinicians to feel confident delivering excellent palliative and end-of-life care, with specialist palliative care available to manage high complexity. Beyond healthcare professionals, initiatives which improve death literacy^4^ (including grief literacy^5^) within society are vital and would strengthen support through social care and for informal carers. The recent growth of death cafés and changes in the workplace such as in-house bereavement support^6^ and bereavement leave entitlement are all positive steps towards normalising discussions about death and grief in society.

## Equity-focused care and shift to community-based services

**Fig. 3:**
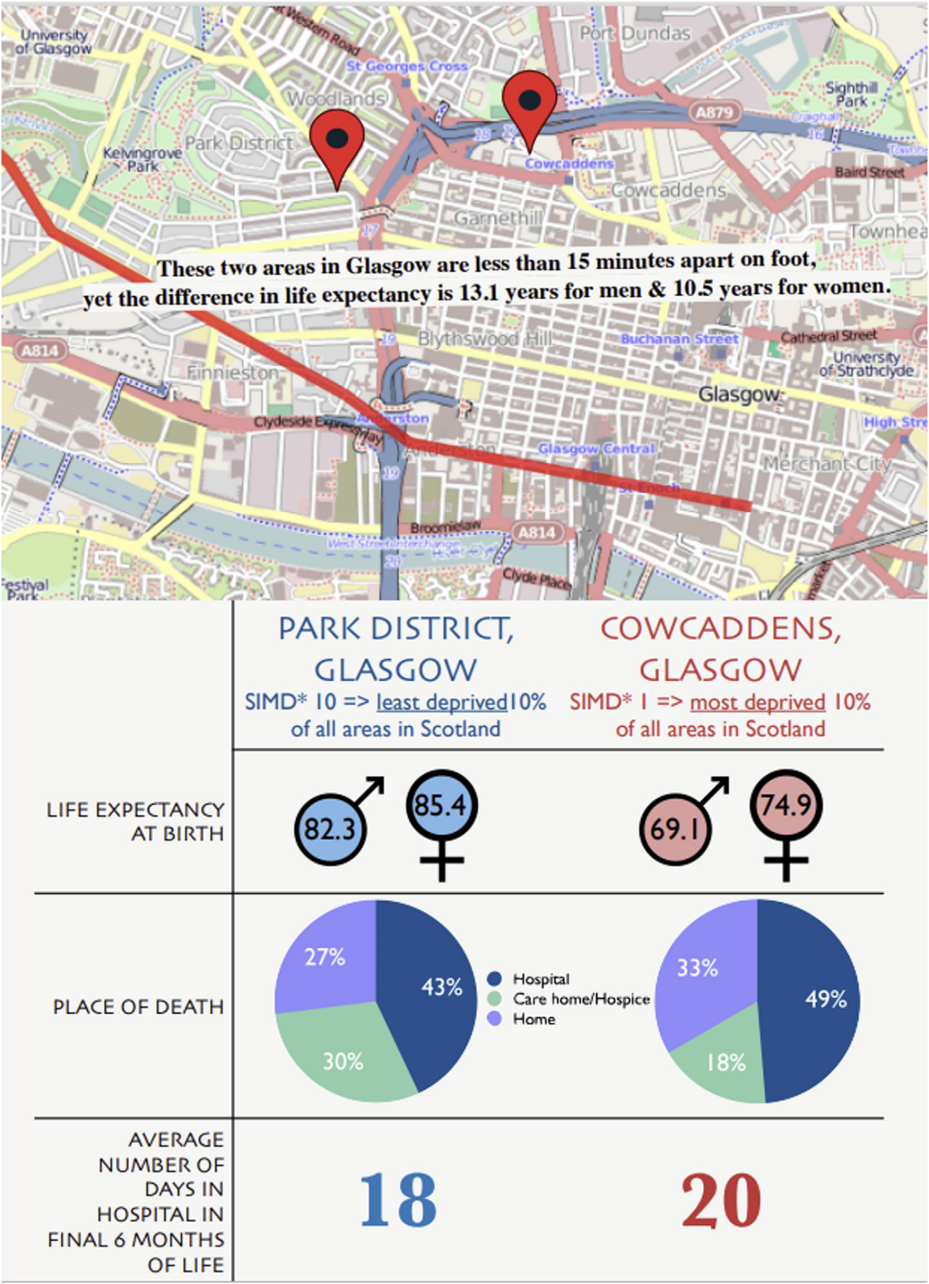
A comparison of life expectancy, place of death and hospital inpatient days in the final 6 months of life, for two neighbouring areas in Glasgow. (*SIMD, Scottish Index of Multiple Deprivation. This is a relative measure of deprivation across Scotland based on income, access to education, health, and services compared to the rest of the country. SIMD 1 (decile 1) refers to the most deprived 10% of areas in Scotland, and SIMD 10 (decile 10) refers to the least deprived 10% of areas in Scotland).^11^, ^12^, ^13^, ^14^, ^15^, ^16^

Evidence suggests that most people would prefer to avoid spending time in hospital towards the end of their lives and to die at home.^8^ Where people die has shifted from the community, with around 50% of UK deaths taking place in hospital, and hospital admissions becoming more frequent in the final months of life. In the year 2026, stark inequities exist in how people spend the end of their lives. For example, the social determinants of health, which put individuals at higher risk of ill health, lead to delayed diagnosis of serious illness and shorter lives. Those living in more socio-economically deprived areas have increased odds of frequent hospital admissions at the end of life and likelihood of death in hospital.^9^ Other aspects of a person’s identity such as race, gender and sexuality also negatively impact on their end-of-life care. These aspects of people’s lives do not exist in isolation; multiple layers of disadvantage and discrimination intersect, bringing further disadvantage.^10^ For example, people living in the UK in the most deprived areas are more likely to die in hospital, but within the most deprived group, being from a minority ethnic group and/or LGBTQ+ further determines inequities and shapes experience of end-of-life care. As we move towards 2050, there is an urgent need to understand intersectional inequalities and how to address these, to have a future where equitable care at the end of life is delivered. Fig. 3 shows an example of socio-economic inequity in the UK today, where people living only a 15-min walk from each other have more than a 13-year difference in life expectancy (for men) and will have different experiences of healthcare, including end-of-life care.

It is important to highlight that admission to hospital can provide meaningful benefit in some situations, and that death in hospital may be preferable for some, especially those facing inequalities where home may not be a safe and secure place.^17^ For many, location is less of a priority than ensuring symptom control and comfort. However, hospital inpatient stays are the most expensive aspect of end-of-life care, with space in hospital a limited resource. It has been identified as a part of the NHS 10 Year Plan^18^ (2025) that it is a priority to move care out of hospitals where possible and into the community. By 2050, the vision would be that significant investment has been made to provide 24-h access to support, to enable people to remain in the community; with acknowledgement that people don’t only have crises during normal working hours. We will see better integration of palliative care with wider services, including bringing hospices fully into the NHS rather than expecting this important aspect of specialist palliative care to be funded by charity.

## Communities

Palliative care did not invent care of the dying. Families and communities have been caring for people dying for as long as humanity has existed. Relationships, connection, meaning and legacy are central parts of dying but are often missing from a clinical response to death and dying, even a multiprofessional palliative care response. While clinical intervention is required, this must sit within a much broader community response. Death and dying are social challenges that require social responses.

**Fig. 4:**
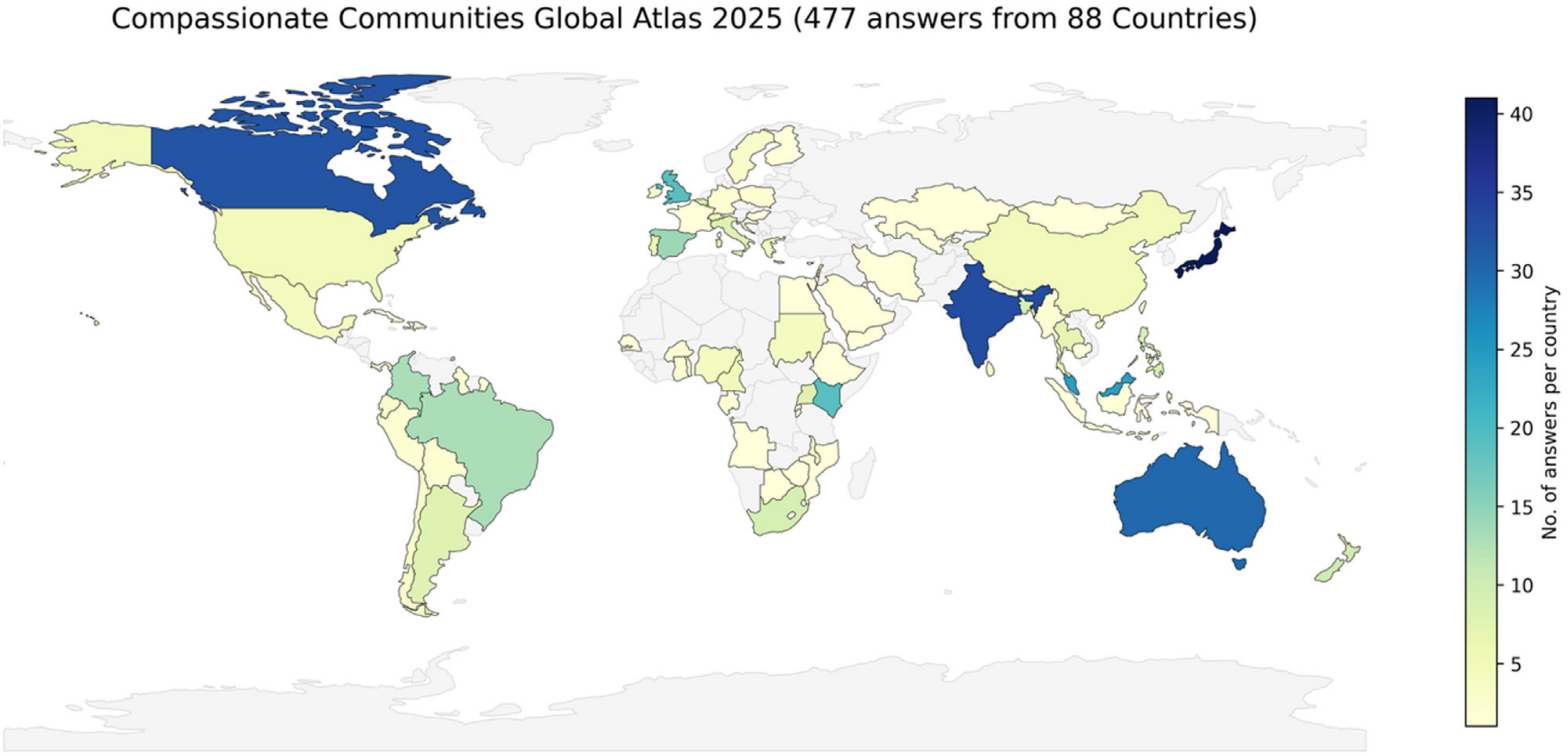
World map of compassionate communities currently – scope for further world growth.^25^

The Compassionate Communities movement is a growing global movement to tackle this challenge. They build confidence in local communities to support those facing the end of life, establish networks, challenge stigma and develop death literacy. Compassionate communities improve wellbeing, social connection and appropriate health service at the end of life.^19^, ^20^, ^21^, ^22^ The first compassionate community was developed in Kerala, India in the 1990s^23^ and a systematic review in 2022 estimated that approximately 15 initiatives existed globally.^24^ Fig. 4 shows the results from a recent global compassionate communities survey with 477 responses across 88 countries.^25^ This explosion of compassionate community initiatives is evidence of a seismic change taking place in care of the dying. If we follow the curve of the trajectory to 2050, we can see a reclamation of care of the dying, with high levels of death literacy, compassionate communities available and accessible to all, working in partnership with clinical palliative care services. Specialists are able to see people with complex needs, on a bedrock of community support, leading to a new and integrated model of care at the end of life.

In addition, it is important to have greater recognition of sources of support already embedded within communities, beyond colleagues in roles traditionally associated with providing palliative care support (such as hospices). With the growing need for social care, more people will be living the last part of their life in care homes and this brings opportunities to improve training for staff and change the culture of palliative and end-of-life care within care homes.^26^ Some care homes have already started by allocating beds specifically for people approaching the end of life, upskilling care home staff and creating key links with wider services. Community pharmacies can also play an important role in education on symptom management and rationalising medications. Additional sources of support such as end-of-life doulas have the potential to also improve care and support within the community.

## Data and research

Despite dying being the only universal health-related population experience, palliative care remains one of the most underfunded areas of healthcare research in the UK. For every £100 spent on medical research, only 26 pence is directed towards palliative care.^27^ Although research outputs have grown fivefold since the early 2000s, even optimistic projections suggest that by 2050, only around 4,500 palliative care-relevant publications will be produced each year.^28^ For context, this is far fewer than the number of oncology-related publications produced each month today.^29^

Historically, palliative research has been constrained by insufficient or fragmented data. However, with integrated electronic health records linked by unique patient identifiers, this limitation is rapidly dissolving. Data linkage can generate insights into hospital admissions, community prescribing, quality-of-life indicators and demographics, enabling a far richer understanding of patients’ lives – and deaths.

These data allow researchers to build predictive models identifying when and who will require palliative input, supporting timely advance care planning,^30^ preventing crises, and highlighting underserved groups, including people with dementia and complex multimorbidity. Defining ‘complexity’ and clarifying who most benefits from specialist input – currently recognised intuitively but poorly measured – are important research priorities.^31^ Referral practices remain inconsistent and often favour individuals who are more health-system literate, with socio-economic inequities leading to those of lower socio-economic position having less access to palliative care.^32^

Finding generalisable solutions for an increasingly heterogeneous population will depend on big data. Large-scale observational resources such as *Our Future Health* – which started recruiting in 2022 – are projected to provide rich, representative data by 2050, including linkage to hospital admission records and death registrations. These datasets can support studies of prognostic markers, frailty indices, dynamic complexity scores and symptom trajectories, and can highlight inequities in referral patterns, symptom control and access across socio-economic, geographic and ethnic lines.

Complementing these observational approaches, genomic datasets and methods such as Mendelian randomisation may increasingly offer alternatives to traditional – and often suboptimal – trials by identifying causal drivers of functional decline, multimorbidity and, in some cases, pharmacological responses.

Together, these advances could enable a proactive, personalised model of palliative care, directing resources where they are most needed and ensuring specialist involvement at the right moment. By 2050, research should begin to make palliative care not only more efficient and evidence-based, but also more equitable.

As death, dying and bereavement have implications beyond healthcare services, it is insufficient to conduct medical research alone to improve experiences towards the end of life. Insights from research combining the social sciences and humanities are vital for understanding aspects such as social and cultural context and meaning.^33^ Greater collaboration between these different research areas is key, to inform policies that improve the experiences of people at the end of life.

## Technology

Technological innovation is transforming healthcare, with digital solutions a cornerstone of the NHS 10 Year Plan.^18^ Although palliative research largely consists of small feasibility studies, digital interventions show potential to improve quality of life and dying. Virtual reality can reduce pain and anxiety and is acceptable to patients.^34^ Wearable and ‘internet of things’ devices enable remote physiological and activity monitoring and may support early identification of deterioration. Telemedicine platforms facilitate remote communication, collection of patient-reported outcomes and shared decision making.^35^ Artificial intelligence (AI) has seen the most rapid adoption, with healthcare-related AI publications increasing more than eightfold between 2017 and 2023.^36^ In palliative care, AI applications have focussed on mortality and symptom prediction, documentation using natural language processing and personalised care planning, though most models are trained retrospectively and lack external validation.^37^ AI is likely to be embedded across electronic health records in end-of-life care, pulling together and analysing data to support clinical decision making.

These developments raise ethical and distributive issues. Digital access and infrastructure mirrors inequities in palliative care and varies with socio-economic status, risking widening existing differences in care provision.^38^^,^^39^ Implementing digital technologies requires extensive data collection and linkage, and questions of consent, access and governance must be addressed in highly sensitive end-of-life contexts. AI-assisted tools informing resource allocation and predicting mortality must be transparent and validated, and accountability must be clearly defined. Training such tools on unrepresentative datasets will entrench existing biases, underscoring the need for diverse, well-governed datasets such as those put forward in the *Our Future Health* initiative.^40^ The limits of AI’s use in palliative care are a point of contention; while AI chatbots are consistently rated as more empathetic than doctors in text-based conversations,^41^ how this translates to sensitive discussions requiring authentic empathy in end-of-life care is unexplored.

## A vision for 2050

In 2050, palliative care has come full circle. The community is once again the place where people are cared for and die, with integrated ecosystems of care at last able to match and meet people’s preferences to live and die at home, or in supported settings close to home. These ecosystems are not restricted to palliative care specialists or healthcare professionals, but are interconnected networks of families, friends, neighbours and community groups working with healthcare workers in primary care and hospital, housing sectors, religious organisations and a host of others who now appreciate their role in supporting people to live and die well.^1^ Crucially, social care is well integrated into this ecosystem.

AI predictive models will play a major role in addressing the inequities that have characterised palliative and end-of-life care services over the preceding years by identifying who needs palliative care, when they need it, and what type of support will be most effective. Digital technologies facilitate unobtrusive remote monitoring of people in the community, allowing those near the end of their lives to remain at home for longer. Using integrated data, including biomedical, patient-reported and social determinants, the challenges with gatekeeping, prognostication, late referrals and stigma will be overcome as palliative care forms an established and unquestioned component of basic care. Precision medicine will transform the selection of pharmacological agents for the management of symptoms, and technological advances such as immersive virtual reality will bring in a new era of non-pharmacological treatments for pain. ‘Grief tech’ will continue to evolve. Avatars, digital immortality and virtual memorials will offer continued connection with those who have died, but bring ethical challenges to address.

Decision-making frameworks, built traditionally around a narrow view of individual autonomy, will expand to acknowledge relational autonomy,^42^ recognising that for many people, choices are not made alone, but in response to and with those important to them. This will reshape advance care planning and how capacity and best interests are understood and enacted at the end of life.

Death will no longer be viewed *only* as failure, but also as a natural and inevitable part of being human. Public health will recognise dying as part of a life-course approach and this phase of life will be subject to the same efforts to promote health and wellbeing as other life stages. Death literacy will be embedded through schooling, workplaces and public health campaigns, helping societies understand these universal experiences and enabling confidence in supporting people at these times.

## CRediT authorship contribution statement

**Donna Wakefield:** Writing – review & editing, Writing – original draft, Visualization, Project administration. **Sarah Perrott:** Writing – original draft, Visualization. **George Turner:** Writing – original draft. **Libby Sallnow:** Writing – review & editing, Writing – original draft, Visualization.

## Declaration of Competing Interest

The authors declare that they have no known competing financial interests or personal relationships that could have appeared to influence the work reported in this paper.
